# Extracting Rhythmic Brain Activity for Brain-Computer Interfacing through Constrained Independent Component Analysis

**DOI:** 10.1155/2007/41468

**Published:** 2007-08-26

**Authors:** Suogang Wang, Christopher J. James

**Affiliations:** Signal Processing and Control Group, ISVR, University of Southampton, Southampton SO17 1BJ, UK

## Abstract

We propose a technique based on independent component analysis (ICA) with constraints, applied to the rhythmic electroencephalographic (EEG) data recorded from a brain-computer interfacing (BCI) system. ICA is a technique that can decompose the recorded EEG into its underlying independent components and in BCI involving motor imagery, the aim is to isolate rhythmic activity over the sensorimotor cortex. We demonstrate that, through the technique of spectrally constrained ICA, we can learn a spatial filter suited to each individual EEG recording. This can effectively extract discriminatory information from two types of single-trial EEG data. Through the use of the ICA algorithm, the classification accuracy is improved by about 25%, on average, compared to the performance on the unpreprocessed data. This implies that this ICA technique can be reliably used to identify and extract BCI-related rhythmic activity underlying the recordings where a particular filter is learned for each subject. The high classification rate and low computational cost make it a promising algorithm for application to an online BCI system.

## 1. INTRODUCTION

The electroencephalogram (EEG) is a recording of the brain's electrical activity and is one of the most important measurements used to evaluate neurological disorders in the clinic and to investigate brain function in the laboratory. The recording is obtained by placing electrodes on the scalp, generally according to the 10/20 electrode placement system [1].

A brain-computer interface (BCI) is a communication system in which messages or commands that an individual sends to the external world do not pass through the brain's normal output pathways of peripheral nerves and muscles [2]. In an EEG-based BCI, the messages are carried through EEG activity. The primary aim is to provide people with a new channel for communication with the outside environment. Many different disorders, such as amyotrophic lateral sclerosis (ALS), brainstem stroke, brain or spinal cord injury, and numerous other diseases can disrupt the neuromuscular channels through which the brain communicates with its environment and exerts control. These kinds of severe diseases may cause people to lose voluntary muscle control and to be unable to communicate in any way (this is known as being “locked in”). As current knowledge about these disorders is rather limited, there are no effective treatments which can provide a cure or even a significant recovery. In the absence of methods for repairing the damage caused by these diseases, a BCI system provides an option that conveys messages and commands to use some devices such as assistive applications and computers. This type of direct brain interface would increase an individual's independence and improve quality of life and also reduce the costs on society.

Historically, EEG activity is divided into four types of continuous rhythmic sinusoidal waves known as *δ*, *θ*, *α*, and *β* frequency bands. In this study, it is the function that allows users to control the amplitude of their *μ* (8–12 Hz) or *β* (18–22 Hz) brain rhythmic activity over the sensorimotor cortices caused by motor imagery (MI) [3, 4] (i.e., hand or foot movement imagination), that is of interest. For MI, the users are instructed to imagine a specific motor action without any related motor output. The imagination of the movement is accompanied by an effect known as event-related (desynchronization/synchronization) (ERD/ERS) [5]. When ERD is present, it is relatively detectable and can be used as a feedback signal to control specially designed electrical devices, for instance, to control the movement of a cursor on a computer screen or to drive/steer a wheelchair. However, imagery is dependent on the individual's ability to generate a good ERD, and hence such a BCI will have variable performance. Moreover, artifacts (such as movement artifacts, eyeblinks, and electrical interference) where they appear change the raw EEG and render the recording virtually unusable.

Many signal processing techniques have been developed and used in BCI studies, such as autoregressive modelling [6], and common spatial patterns [7]. These methods tend to find a spatial filter to maximally improve the signal noise ratio (SNR). In order to reach an optimal performance, some additional processing methods are required as preprocessing steps before the application of, for example, bandpass filtering, common average reference, or manual artifact rejection. A combination of preprocessing methods could improve the performance, but also results in a less flexible and robust BCI system. Moreover, the application of more additional processing methods brings with it the problem of increased computation time.

Blind source separation (BSS) techniques such as Independent component analysis (ICA) have the ability to extract the relevant information buried within noisy signals and allow the separation of measured signals into their fundamental underlying independent components (ICs). Generally, the signal is assumed to be a linear mixture of statistically independent, possibly nonstationary sources which may be decomposed using either statistical and information theoretic signal properties (such as the popular method of fast ICA [8] and infomax ICA [9]), or signal time structure (time-structure-based ICA) [10]. ICA has already been quite broadly applied to the analysis of biomedical signals, such as analysis of EEG [11], ECG [12], MEG [13], and fMRI [14].

Recent studies have applied ICA in BCI applications [15–17]. The results indicate that ICA performed well in extracting *time-locked* features, such as event-related potentials (ERPs). However, since MI-based BCI does not use time-locked activity but rather relies on rhythmic activities as features, traditional applications of ICA are unable to track the changes in power spectra among the different sources. Using time-structure-based source decomposition methods, we can capture the sources with stationary waveforms and unique power spectra. Furthermore, when the power spectrum of the particular source activity is known, the spatial extent of the sources can be extracted by introducing *a priori* constraint(s) through constrained ICA (cICA). Our previous studies where we extracted rhythmic EEG signal components (such as epileptic seizures) have been shown in [18, 19].

In this work, we examine the use of existing cICA algorithms that we have previously developed to extract reliable spectral features in the BCI paradigm of MI. The ultimate aim of applying cICA is to extract rhythmic scalp EEG activity automatically and repeatedly from the recorded signals, so that the MI-based BCI system is more reliable and robust —especially for use outside of the clinical laboratory (i.e., in the presence of artifacts and across different subjects). In the following sections, we describe the cICA algorithm, the selection of power features from the datasets, and the overall classification system used. We then present the results obtained and discuss the performance enhancements to be achieved from the use of this algorithm.

## 2. METHODS

### 2.1. Independent component analysis

In the standard, noise-free formulation of the ICA problem, the observed signals **x**(*t*) are assumed to be a linear mixture of an equal number of unknown but statistically independent source signals **s**(*t*):

(1) x(t)=As(t),

where the square mixing matrix **A** is also unknown but invertible. The columns of **A** each depicts a spatial topography for each of the ICs in **s**(*t*). The problem is solvable up to a permutation, and sign and power indeterminacy of the sources, by finding an appropriate de-mixing matrix **W** = **A** ^−1^ which allows estimation of the source waveforms by

(2) s(t)=Wx(t).

Source decomposition on the basis of signal time structure may be achieved through temporal decorrelation (TD). For sources with stationary waveforms and unique power spectra, the time structure is adequately captured by temporal cross-covariances [20, 21]. The decorrelation operation in time structure ICA methods involves the joint diagonalization of a set of symmetric matrices which reflect the spatio-temporal covariance structure of the source mixture. Furthermore, algorithms have recently been developed for nonorthogonal joint diagonalization that processes signal covariances directly with no need for prewhitening, one such algorithm is used here and is called LSDIAG [22].

Assume that there is a set {**C**_1_,…,**C**_*k*_} of real-valued symmetric matrices, the TD approaches find a transformation **W** that in some sense diagonalizes all the given matrices based such that

(3) $${\text{C}}_{\tau }^{s}=\text{W}C_{\tau }^{x}{\text{W}}^{T}$$

for time lags *τ* = 1, 2, 3,…, where $C_{\tau }^{x}$ is the signal covariance matrix and $C_{\tau }^{s}$ is source covariance matrix. Estimation of **W** reduces to the well-researched problem of joint (approximate) diagonalization of the stack of matrices given by $\text{W}{\text{C}}_{\tau }^{x}{\text{W}}^{T}$, for which a fast and efficient new algorithm LSDIAG_TD_ is used.

### 2.2. Constrained ICA

Once a set of sources is determined through ICA, the ICs of interest must be identified. This is made difficult as the nature of the square mixing matrix means that a great many more sources will be identified over the expected (smaller) number of sources underlying the measurement set. A practical way to extract only the sources of interest automatically is to use prior knowledge or additional constraints on the source model—cICA—through the use of a constraint or reference vector. The reference vector can be any vector which incorporates appropriate prior knowledge into the system. In this work, as we are interested in rhythmic EEG signals within our EEG recordings (specifically *μ*-rhythm activity), we propose to use a predefined spectral reference as the constraint. This spectral constraint then allows only those source activities with the same power spectrum to be extracted via the cICA algorithm. In [23], our innovation was to include a reference channel added as an extra row to the measurement matrix **x**(*t*), such that a new matrix $\hat{x}(t)$ is created with

(4) $$\hat{\text{x}}(t)=\left[\begin{array}{c} \text{x}(t)\\ {\text{c}}_{1}(t) \end{array}\right],$$

where **c**_1_(*t*) is a suitable reference vector. In order to observe changes in rhythmic activity in specific frequency bands, we use band-pass- (BP-) filtered white noise to derive a reference signal. Particularly, we use an 8th order Butterworth BP filter with lower and upper corner frequencies set appropriate to the desired constraint. The ICA problem is now such that the extra row in the measurement space due to the reference vector results in an extra row in the IC space after the ICA step (as well as a corresponding extra column in the mixing matrix). For an *n*-channel system, the first *n* elements of the extra mixing matrix column [${\text{a}}_{1}^{n+1},{\text{a}}_{2}^{n+1},\ldots ,{\text{a}}_{n}^{n+1}$] depict the spatial distribution (topography) of the new IC given by the row vector **s** *n* + 1(*t*). Furthermore, each of the elements of the (*n* + 1)th row of the mixing matrix reflects a weighting of each corresponding IC. This row vector, **a** *_n_* + 1, can in fact be used to depict the contribution of each topography described by the columns of the mixing matrix, due to the reference channel **c**_1_(*t*). In this way, ICA now provides the desired convenient spanning basis, and can also be used to obtain the topography of interest (extracted by summing the weighted contribution of each column of the mixing matrix). Furthermore, the weighting value of each IC provides us with a spectrum of values that can be interpreted to gain some insight into the complexity for a given reference. The above technique can be readily extended to more than one reference. However, in this work we apply the method using just a single (*μ*-rhythm) reference. Some techniques, such as this, have already been included in the popular free ICA toolbox—ICALab [24].

### 2.3. The reference channel

Since the phase information of this added reference channel is meaningless (i.e., we cannot expect the phase of the reference signal to be connected in any way to that of the desired brain response), we overcome the problem of matching the phase of the reference channel with that of the desired activity in the recordings, through calculating the lagged covariance matrices that LSDIAG requires via the fast Fourier transform (FFT) and then removing the phase information of the signal in the frequency domain. Recall that the cross-correlation of two functions *f*(*t*) and *g*(*t*) can be obtained through convolution of *f* and *g*, such that

(5) $$f*g=F^{-1}[\bar{F}(v)G(v)],$$

where ∗ denotes convolution, *F* ^−1^ denotes the inverse Fourier Transform, *F*(*v*) and **G**(*v*) denotes the Fourier transform of *f* and *g*, respectively, and $\bar{F}(v)$ denotes the complex conjugate.

### 2.4. The dataset

In this work, we used two datasets: the 2003 BCI competition dataset IIa (self-regulation of *μ*- and/or central *β*-rhythm) and the BCI competition III dataset IVa (motor imagery, small training sets) which are obtainable from ida.first.fraunhofer.de/projects/bci/competition–,ii and competitioniii. In dataset IIa, the subjects either increase or decrease their *μ*- or *β*-rhythm amplitude power to control a cursor's vertical position aiming to the height of the correct target through visual feedback. In dataset IVa, the subjects imagine either right-hand or right-foot movements indicated by a visual cue on-screen without feedback. Although these two experiments were designed in different ways, they both used the property of ERD power spectrum adjusted by different specific activation.

**Dataset 1**

The 2003 BCI competition dataset IIa (self-regulation of *μ*- and/or central *β*-rhythm) was used, which was provided by the Wadsworth Center [25]. This dataset contains a whole record of an actual BCI system from 3 trained subjects in 10 sessions (about 30 minutes per session) each. EEG was recorded from 64 scalp electrodes (10/20 system) sampled at 160 Hz. For this BCI to work, after a one-second resting period during which the screen stays blank, a target appears at one of four possible positions on the right-hand side of the screen. One second later, a cursor appears at the middle of the left of the screen and starts moving at constant speed across the screen from left to right. When the cursor reaches the right-hand side, the screen is cleared and the next trial begins. The experiment includes visual feedback whereby the vertical position of the cursor on the screen is determined through brain activity. Three data subsets marked as AA, BB, and CC are supplied. Each session consisted of 192 trials (48 trials for each target can be “top,” “upper middle,” “lower middle,” or “bottom”). The first six sessions are labelled as *training* sets. The remaining four sessions are *test* sets and not labelled initially for the purposes of the competition. After the competition, the labels for testing sets were released and the datasets become available for developing new methods towards improving BCI studies. In this work, we only select trials with the target position code: “top” (Target 1) and “bottom” (Target 2) to examine our proposed method.

**Dataset 2**

The BCI competition III dataset IVa from the Berlin BCI group [26] was used. This dataset contains 118 multi-channel (extended 10/20 system) EEG signals recorded from five healthy subjects (labelled “aa,” “al,” “av,'' “aw,” and “ay,” resp.) at a sampling rate of 100 Hz. During the experiments, subjects were prompted by a displayed letter (R/right hand, or F/right foot) to imagine for 3.5 seconds either right-hand (Target A) or right-foot movements (Target B) without feedback. Each type of MI was recorded 140 times, thus in total there are 280 trials for each subject. Between the trials, there was a random period of time (1.75 to 2.25 seconds) in which the subject could relax. This dataset also brings with it a challenge in that only a little amount of training data are available, this allows us to examine the influence of using small training sets in order to reduce the training time. The task is to classify the type of the imagined movement for each trial in an offline fashion.

### 2.5. The proposed algorithm

**Figure 1:**
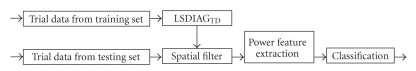
A diagram depicting the proposed algorithm. It includes three parts: spatial filter generation, power feature extraction, and classification.

The algorithm we propose includes three parts: (a) spatial filter generation, (b) power feature extraction, and (c) classification. This is depicted in diagrammatic form in Figure 1.

#### 2.5.1. Spatial filter generation

For the analysis, a number of epochs of the training dataset were used to estimate the lagged covariance matrix stack $C_{\tau }^{x}$. We treat the stack of matrices as arising from two-part averaged lagged covariance matrix stacks $C_{\tau }^{XT_{1}}$, $C_{\tau }^{XT_{2}}$ in which each part is obtained from trial data corresponding to one of two targets, such that

(6) $$\begin{array}{c} {\text{C}}_{\tau }^{x}=[{\text{C}}_{\tau }^{XT_{1}};{\text{C}}_{\tau }^{XT_{2}}],\\ {\text{C}}_{\tau }^{XT_{1}}=[\frac{1}{m}\sum_{x_{k}\in XT_{1}}{\text{C}}_{0}^{x_{k}},\ldots ,\frac{1}{m}\sum_{x_{k}\in XT_{1}}{\text{C}}_{l}^{x_{k}}],\\ {\text{C}}_{\tau }^{XT_{2}}=[\frac{1}{n}\sum_{x_{k}\in XT_{2}}{\text{C}}_{0}^{x_{k}},\ldots ,\frac{1}{n}\sum_{x_{k}\in XT_{2}}{\text{C}}_{l}^{x_{k}}], \end{array}$$

where *τ* = [0,…, *l*] depicts the range of lags (here *l* = 5 as determined in previous work [27]). *x* ∈ [*X T*_1_,*X T*_2_] denotes that trial data are from training set corresponding to the labels: Target 1/A and Target 2/B. The number of trials in each dataset, *X T*_1_ and *X T*_2_, is *m* and *n*, respectively. Here we set the value of *m* equal to *n* to balance the proportion of trials for both targets.

The spectrum, **P**(*i*), is defined as a trial spectrum in *i*th channel by the sum of the ordinates of the frequency bins (**h** *d*) within the proposed frequency band, that is,

(7) $$\text{P}(i)=\sum_{d\in D}{\text{h}}_{d},$$

where *D* denotes the number of frequency bins. After cICA decomposition, the EEG data are extracted into the ICs. Thus, the power spectrum after cICA is defined as the sum of the weighted spectra of sources (ICs) within the *μ* band. So, for given source epochs, the power feature reflected in an individual channel is defined as

(8) $$f_{p}(i)=\sum_{j=1}^{k}a_{k+1,j}P_{ic}(j)a_{i,j},$$

where *k* denotes the number of sources. As this implementation of cICA assumes a square mixing matrix, then the number of sources is the same as the number of measurement channels, and **a** *_i, j_* is an element in the mixing matrix **A**. **a**_*k*+1, *j*_ is a particular element in the last row of **A**. **P** *_ic_*(*j*) denotes a trial spectrum in the *j*th IC source.

#### 2.5.2. Feature selection

**Figure 3:**
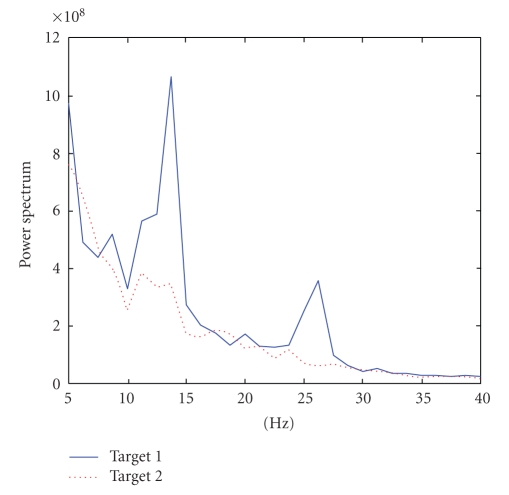
Averaged power spectra of trials corresponding to Target 1 and Target 2 (Subject “CC”). In this experiment, greater power (Target 1) implies the cursor going up and vice versa.

**Figure 4:**
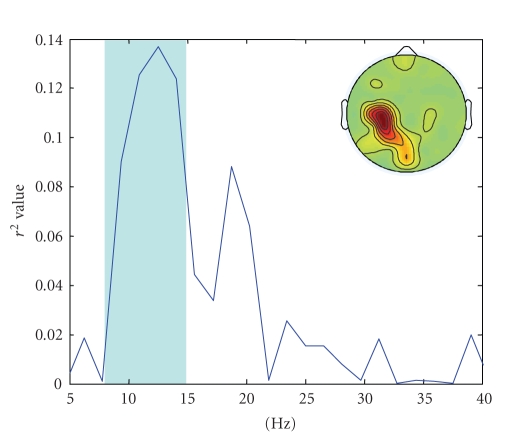
*r* ^2^ values across the spectrum on C3 channel for Target A and Target B (Subject “ay”). The shadowed frequency band was chosen in this work. Inset is the topography of the *r* ^2^ values at 12.25 Hz across channels.

**Figure 5:**
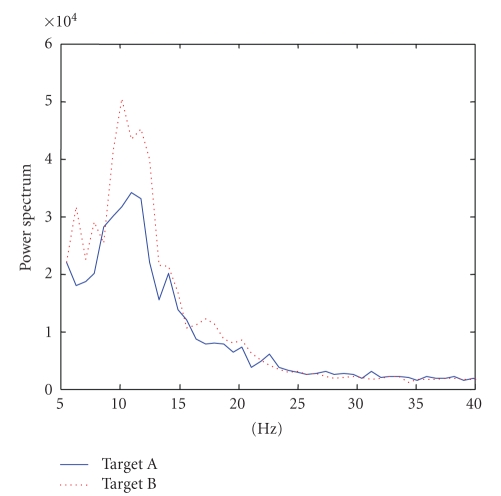
Averaged power spectra of trials corresponding to Target A and Target B (Subject “ay”). The averaged power for imagined foot movement (Target A) is greater than the power for hand movement imagination (Target B).

In order to find discriminative power bands for each subject, we calculated the power spectra of two targets in these two datasets, and then combined the variables on each individual channel into *r* ^2^ values which represent the proportion of the variance of spectral power values from the labelled training sets. By comparing to the averaged power spectra corresponding to two targets, this describes the relationship between power intensity and target labels. These parameters were slightly different due to differences in each individual recording. For example, in dataset IIa, two discriminative power bands roughly around 10–15 Hz and 23–28 Hz (Figure 1) are used. In this work we chose 10–15 Hz as the working band. Increased power is taken to correspond to Target 1 which raises the cursor in Subject AA and Subject CC while it makes the cursor go down in Subject BB (Figure 3). In dataset IVa, we selected the subband approximately around 8–15 Hz to calculate power (Figure 4). Increased power is related to Target 2 which is the right-foot imagination (Figure 5) in all subjects.

As described in the above section, the data were originally recorded from 64 scalp electrodes for dataset IIa and 118 electrodes for dataset IVa. We are only interested in the activity in the motor cortex, so the electrodes around the sensorimotor cortex were chosen manually, these included C5, C3, C1, C2, C4, C6, CP5, CP3, Cp1, Cp2, Cp4, Cp6, P5, P3, P1, P2, P4, and P6. We only used a small segment of EEG data for training in the proposed algorithm: for dataset IIa, the data between 0.5–2 seconds of each trial are used after the cursor is displayed on the screen; for dataset IVa, the data between 0.5–2.5 seconds are considered after the instruction is displayed on the screen.

#### 2.5.3. Classification

In order to evaluate the performance of the proposed algorithm, we only consider a simple one-dimensional linear classifier based on thresholding the power feature(s) in the chosen frequency band for the final classification. The threshold value is selected by minimizing the number of trials misclassified in both classes from the training set for individual subjects. In addition, as a comparison for the classification performance, we also applied a more complex classifier, a support vector machine (SVM) [28] which constructs a nonlinear separation hyperplane based on a machine-learning algorithm.

The next procedure is to decide which power feature will be suitable to use for the classification. Based on the distribution of *r* ^2^ values across the topography maps in the previous section, a number of channels (between 1 and 3) around the left sensorimotor cortex were selected. The power on C3 was used in the threshold classifier and the power on C3, CP1, and CP5 for the SVM classifier as the use of these power features was found to be able to achieve better classification accuracy in our study.

## 3. RESULTS

Using the proposed method, the designed spatial filters will be able to capture the relevant dynamics of the subject's brain state more robustly. Furthermore, the resulting time series would have optimized the spectra which could result in better discrimination between two different brain states. The results show that following this pre-processing, even a simple linear classifier can achieve superior classification accuracy.

**Figure 6:**
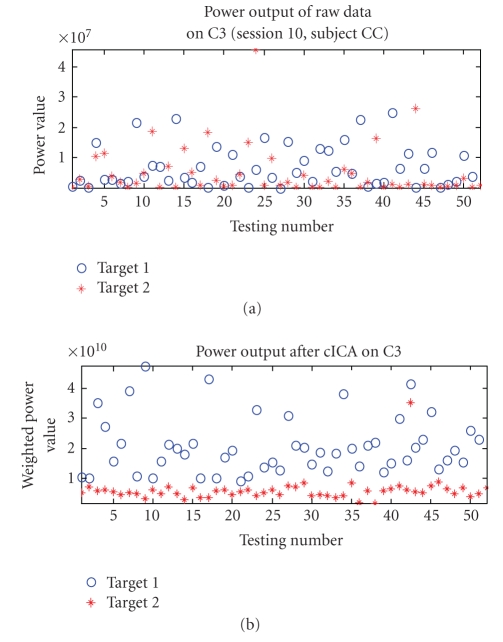
The power feature outputs of Subject CC for Testing session 10. (a) shows the power features on C3 using unprocessed data; (b) shows the power features on C3 after cICA processing. A circle denotes Target 1 (drive cursor up); a star indicates Target 2 (cursor down).

**Figure 7:**
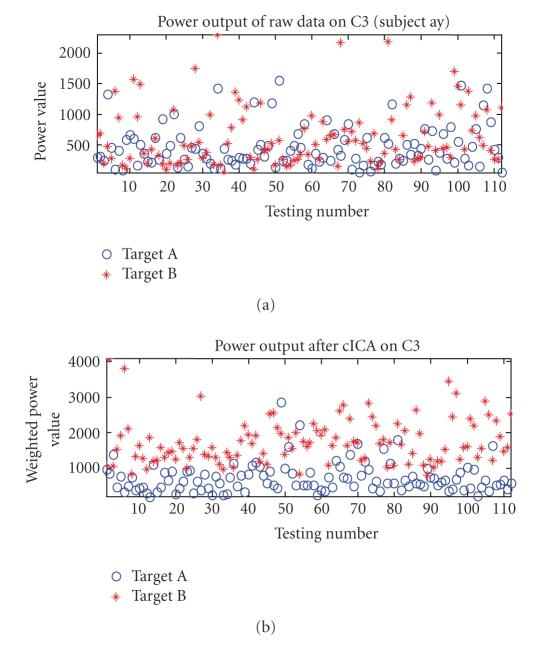
The power feature outputs for Subject “ay” on testing set. (a) shows the power features on C3 using unprocessed data; (b) shows the power features on C3 after cICA processing. A circle denotes the power feature for Target A (right hand imagination); a star indicates the power feature for Target B (right foot imagination).

Figures [6 and 7] depict the power features related to different targets before and after the processing for channel C3. In Figure 6, we plot the power features of testing session 10 for Subject CC in dataset IIa. Ideally, the higher power feature is for Target 1 and lower power for Target 2 (Figure 3). However, without spatial filtering, the power features between two targets from the original data appear overlapped, and a classifier based on either a simple linear method or a potentially complicated advanced method is hardly able to separate these patterns efficiently. After the cICA processing, the weighted power values for two different targets are more widely separated than the power features from the unprocessed data. Figure 7 shows the power features of Subject “ay” from dataset IVa. The power related to the right-hand movement imagination is marked as Target A and the power for right-foot movement imagination is marked as Target B. As shown in Figure 5, the averaged power for imagined foot movement is larger than the power for hand movement imagination, but powers correlated to two different targets do not show much different in the raw data. After processing, the power features are maximally separated into the different levels, which further demonstrate the improved separation achieved by using this spatial filter. The above examples suggest that the use of this spatial filter can help to extract different brain activities within a particular *μ* rhythmic band.

Table 1 lists the classification results on the test sets (most sessions have 52 trials for each target, several have 51 trials) in dataset IIa. For each subject, we use 80 randomly chosen trials in total (40 for each target) to calculate the spatial filter. The results are shown as three columns for each individual subject. The first column shows the results using the unprocessed data. The results of using a threshold-based classifier with one power feature on C3 are shown in the second column. The third column is for the results from an advanced SVM-based classifier using three power features on C3, CP1, and CP5. Table 2 shows the classification performance on the testing data in dataset IVa. There are five subjects contributing to individual subsets with different sizes of training and testing sessions. The numbers of available trial data for training/testing sessions are shown in the first column. To construct the spatial filter, the total number of training trials is selected between 28 and 80 (average of 65 trials was used) due to the different size of training sets. As before, one power feature on C3 is used for the final classification based on a threshold and a linear classifier. Moreover, three features on channel C3, CP1, and CP5 were also applied to examine the performance of an SVM classifier. In addition, as a comparison, the last column lists the classification results from previous published work [29] which proposes a method based on dynamical system (DS) features together with an SVM classifier. The overall classification accuracy is about 85% by this DS+SVM method. From the two tables, we can see that cICA implementation extracts the related rhythmic information very effectively. After processing, the classification accuracy was of 82% for Subject AA, 69% for Subject BB, and 90% for Subject CC in comparison with the average 62% accuracy before processing in dataset IIa. In dataset IVa, the classification accuracy was of an average of 82% through five testing sets, which is 30% higher than the accuracy using the unprocessed data. It is worth noting that the more advanced SVM-based classifier did not show a significant improvement in performance on the same data, although an increase of about 2% compared to the simple linear classifier was observed.

## 4. DISCUSSION AND CONCLUSIONS

Two datasets have been used to examine the performance of the proposed algorithm. Dataset IVa concerns MI data, and dataset IIa regards the self-regulation of *μ*/*β*-rhythm data. Both of these datasets use the characteristic that changes in the amplitude of sensorimotor rhythms over the right/left hemisphere act as the major control pattern. The difficulty here is to maximally and reliably identify two classes from single-trial data. The proposed ICA technique using constraints has been developed and applied to isolate and extract the power spectrum in the rhythmic band of interest. In order to demonstrate the performance of the proposed cICA, we only applied the power feature in the *μ*-rhythm frequency band as the major classification pattern. The results, using a simple linear classifier and an advanced SVM to classify the ICA processed data, show that the classification accuracy has considerably increased over processing the raw data. After the basic analysis, the overall classification accuracy is improved about 20% in dataset IIa and 30% in dataset IVa. As an additional comparison of classification performance to cICA in dataset IVa, we cited the results of a method using DS features as well as an SVM in a publication. This method also includes two steps for data pre-processing (an identical temporal filter and a spatial filter). The accuracy was about 3% more than the results of cICA with a linear classifier and 1% more than cICA with an SVM. If we use a set of well-tuned parameters to the proposed method, then the classification would be expected to reach a slightly better performance. Furthermore, the use of a linear classifier following a simple spatial filter as in our system is desirable from a computational complexity perspective.

As this work is an application to single trial classification, the sensitivity to artifacts in the EEG becomes a major problem. The LSDIAG ICA algorithm uses the covariance of the trail data to estimate the covariance stack matrices which are the essentials to calculate the unmixing matrix and hence the spatial filter. The random selection of training trials with artifacts can cause serious changes to the final filter. Therefore, most methods require that the data should be artifact-free, which can be achieved by several preprocessing steps such as filtering or manual artifact rejection. Here, instead of applying any preprocessing methods before hand, we estimate the stack matrices by using the averaged lagged covariance matrices from the data. The idea behind the process is that the influence of artifacts is reduced since the procedure of averaging the covariance matrices acts as a filter which could balance and minimize the random noise level. Moreover, the system includes a training phase used to tune the proper unmixing matrix (spatial filter) using the proposed ICA. Once the unmixing matrix has been computed, it works as a spatial filter to remove the additional artifacts by weighted spatial averaging the testing data trials and returns the processed time series patterns. After filtering, the different brain activities in the form of power can be clearly extracted. It indicates that through the use of cICA, it is possible to track the rhythmic changes of different brain states in the EEG. These results show a clear improvement for use in this kind of BCI system.

In order to bring a BCI system to work outside of laboratory conditions, several items need to be taken into account in future work. The number of electrodes used in the system usually decides the cost of hardware and the related difficulty of processing the ensuing data. The application of ICA using fewer channels or even a single channel may be the solution of the problem and is one area we are actively pursuing [30]. Similarly, classification pattern selection may be improved as the use of similar patterns (even if the features are from different channels) might limit the capability of the classifier so that even this advanced method cannot work most effectively. Therefore, careful selection of diverse features may alleviate the problem, that is, features in time or in different frequency bands, and so forth. This may further improve classification accuracy.

## Figures and Tables

**Figure 2:**
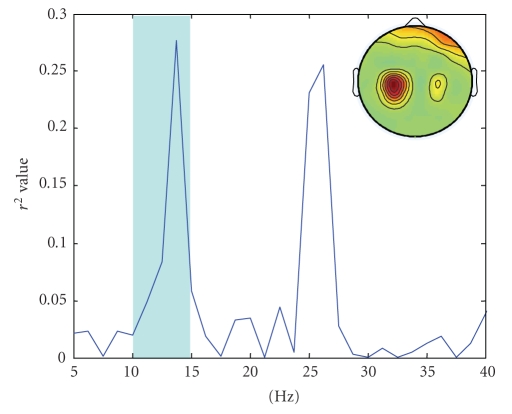
*r* ^2^ values across the spectrum on C3 channel for Target 1 and Target 2 (Subject CC). The shadowed frequency band was chosen in this work. Inset is the topography of the *r* ^2^ values at 13.75 Hz across channels.

**Table 1:** Classification accuracy of the test set based on power feature(s) in dataset IIa. The three columns for each individual subject show the performance of linear classification on unprocessed data, linear classification and SVM classification on the processed data.

Testing dataset	Data AA			Data BB			Data CC		
	
	Linear classifier on raw data	Linear classifier on extracted data	SVM on extracted data	Linear classifier on raw data	Linear classifier on extracted data	SVM on extracted data	Linear classifier on raw data	Linear classifier on extracted data	SVM on extracted data
Set 7	64.6%	80.2%	85.4%	65.6%	72.0%	73.0%	58.3%	85.4%	87.4%
Set 8	59.4%	88.5%	89.6%	71.9%	72.9%	72.9%	62.1%	92.2%	90.3%
Set 9	61.5%	80.2%	79.2%	66.8%	63.5%	67.7%	60.1%	86.1%	88.1%
Set 10	65.6%	80.2%	80.2%	59.4%	68.8%	72.9%	61.2%	96.1%	98.1%

**Table 2:** Classification accuracy of the testing set based on power feature(s) in dataset IVb. The columns depict the results using the three proposed classification schemes, and the last column lists published [29] for comparison.

dataset	Training/test trials	Linear classifier on raw data	Linear classifier on extracted data	SVM on extracted data	SVM on DS features
al	224/56	48.2%	85.7%	89.3%	96.3%
aa	168/112	46.0%	83.0%	85.7%	83.3%
av	84/196	49.5%	75.0%	75.0%	72.7%
aw	56/224	55.4%	80.3%	85.3%	86.9%
ay	28/252	54.3%	85.0%	85.0%	89.0%
